# Comparative vasorelaxant effects of atorvastatin and rosuvastatin in rat aorta: investigating the role of perivascular adipose tissue

**DOI:** 10.55730/1300-0144.5996

**Published:** 2025-02-26

**Authors:** Deniz KALELİ DURMAN, Meryem ARAS, G. Ruveyda AKTAŞ, B. Sönmez UYDEŞ DOĞAN

**Affiliations:** 1 Department of Pharmacology, Faculty of Pharmacy, İstanbul University, İstanbul, Turkiye; 2 Graduate School of Health Sciences, İstanbul University, İstanbul, Turkiye; 3 Department of Pharmacology, Faculty of Pharmacy, Biruni University, İstanbul, Turkiye

**Keywords:** Atorvastatin, rosuvastatin, perivascular adipose tissue, vascular relaxation

## Abstract

**Background/aim:**

Statins are known to display pleiotropic effects on the vascular system, beyond their lipid-lowering properties. Studies on isolated vascular preparations have demonstrated their acute relaxant effects on vascular tone. Considering the increased evidence in regard to the contribution of perivascular adipose tissue (PVAT) in the regulation of vascular homeostasis, we aimed to investigate the possible modulatory role of PVAT in the vascular effects of atorvastatin and rosuvastatin in isolated rat aorta.

**Materials and methods:**

Thoracic aortas isolated from young male Wistar rats were divided into two groups: rings with intact PVAT (+) and rings without PVAT (−), and then mounted in an isolated organ bath system. Rat aortic rings were standardized with potassium chloride (KCl, 40 mM), and then endothelium-dependent relaxation responses were checked by acetylcholine (Ach, 10^−7^–10^−4^ M). The concentration-dependent (10^−7^–10^−4^ M) effects of atorvastatin and rosuvastatin were studied on rat aortic rings precontracted submaximally with phenylephrine (Phe, 10^−6^–3 × 10^−5^ M). In addition, endothelium-independent relaxation responses were evaluated by sodium nitroprusside (SNP, 10^−6^ M) at the end of each experiment.

**Results:**

Rat aortic rings with intact PVAT (+) and without PVAT (−) displayed similar endothelium-dependent and -independent relaxations to Ach and SNP, respectively. Increasing concentrations (10^−7^–10^−4^ M) of atorvastatin and rosuvastatin directly relaxed the aortic rings with and without PVAT. The maximum relaxant effects of rosuvastatin was found significantly greater than atorvastatin.

**Conclusion:**

The current study demonstrated that atorvastatin and rosuvastatin displayed prominent relaxations in rat aortic rings with intact PVAT (+) and without PVAT (−). Notably, rosuvastatin produced a greater vasorelaxant effect compared to atorvastatin in rat aortic rings with and without PVAT. Current study provides a novel evidence that PVAT does not significantly influence statin-mediated vasorelaxation under physiological conditions.

## 1. Introduction

Statins, primarily recognized for their lipid-lowering properties, display several pleiotropic effects including antioxidant, anti-inflammatory and antithrombotic actions [1–5]. Notably, statins are shown to produce relaxations in various vascular preparations by endothelium-dependent and -independent mechanisms [6–15]. Although, previous in vivo and in vitro studies documented that statins have broader biological effects on vascular function beyond lipid modulation, there is no in vitro evidence on how perivascular adipose tissue (PVAT) influences the direct vascular effects of these drugs.

PVAT, which is a dynamic tissue surrounding blood vessels, plays an important regulatory role in vascular tone and homeostasis. The presence of PVAT is shown to attenuate contractions to various spasmogens including noradrenaline, serotonin, angiotensin II, and phenylephrine by releasing vasoactive substances that influence vascular function [16–19]. Moreover, statin treatment, particularly atorvastatin, was shown to enhance the anticontractile properties of PVAT on rat aortic rings in healthy and pathological conditions [20–21]. In addition, in vivo atorvastatin treatment in hyperlipidemic and hyperglycemic animal models has been shown to ameliorate the inflammatory profile of PVAT [22–24]. However, the precise role of PVAT in modulating the vasorelaxant effects of statins remains unclear.

This study aims to investigate the potential influence of PVAT on the direct vascular relaxant effects of atorvastatin and rosuvastatin, the most potent statins, which exhibit comparable efficacy in patients with coronary artery disease in terms of cardiovascular outcomes [25]. For this purpose, the concentration-dependent (10^−7^–10^−4^ M) relaxant effects of atorvastatin and rosuvastatin were examined in isolated rat thoracic aortic rings with intact PVAT (+) and without PVAT (−).

## 2. Materials and methods

Young male Wistar rats (8–10 weeks old, ~250 g) were obtained from Istanbul University, Aziz Sancar Institute of Medical Sciences, Experimental Animals Laboratory (DETAE), and maintained at 21 ± 2 °C, 45%–65% humidity, and a 12 h light/dark cycle, with food and water provided ad libitum. All experimental protocols were carried out in the Experimental Animal Care and Research Unit of Istanbul University Faculty of Pharmacy (EDEHAB), according to the approval of the Local Ethics Committee of Animal Experiments of Istanbul University (IU-HADYEK) (22.11.2023, No: 2023/35-2267678). The animals were housed in the EDEHAB for one week of acclimatization to be accustomed to the environment and then included in the experiments.

The rats were anesthetized via intraperitoneal (i.p.) injection of ketamine (100 mg/kg) and xylazine (10 mg/kg). The thoracic aortas were immediately excised and placed into a Krebs Ringer-bicarbonate solution containing (mM): KCl 4.7, NaCl 118, NaHCO_3_ 25, KH_2_PO_4_ 1.2, MgSO_4_.7H_2_O 1.2, CaCl_2_ 2.5, glucose 10 and disodium EDTA 0.026. Isolated thoracic aorta was divided into 3–4 mm rings, with PVAT left intact in PVAT (+) group, whereas it was removed in PVAT (−) group. The isolated rat aortic rings were then immediately transferred to the laboratory for vascular reactivity studies using the isolated organ bath system.

Isolated rat aortic rings with and without PVAT (+/−)were mounted in an isolated organ bath system containing Krebs-Ringer bicarbonate solution, maintained at 37 °C, and aerated with a mixture of 95% O_2_ and 5% CO_2_. The contractile and relaxant responses were recorded by a force-displacement transducer on a polygraph system (PowerLab-ADInstruments, Oxford, UK). The isolated organ bath experiments were conducted as described previously [6].

### 2.1. Experimental protocol

The aortic rings were equilibrated for 1 hour under a resting tension of 1 g. Afterwards, two consecutive contractions were conducted with potassium chloride (KCl, 40 mM) to standardize the aortic rings, and tissues that produced a contraction of less than 0.5 g were discarded. The functional integrity of the endothelium was assessed by the cumulative administration of acetylcholine (Ach, 10^−7^–10^−4^ M), an endothelium-dependent vasodilator, to aortic rings that were submaximally (70%–80%) precontracted with the selective α_1_-adrenergic agonist, phenylephrine (Phe, 10^−6^–3 × 10^−5^ M). The vasorelaxant capacity of the aortic rings was further evaluated at the end of each experiment by administering sodium nitroprusside (SNP, 10^−6^ M), a directly acting nitrovasodilator.

The experimental protocol aimed to investigate the vascular effects of atorvastatin and rosuvastatin on rat aortic rings with intact PVAT (+) and without PVAT (−), which were submaximally precontracted with Phe (10^−6^–3 × 10^−5^ M). When Phe-induced contractions reached a stable plateau, atorvastatin and rosuvastatin were applied cumulatively at increasing concentrations (10^−7^–10^−4^ M). To analyse the possible effect of the vehicle for atorvastatin and rosuvastatin, DMSO (<0.1%) was applied cumulatively. Additionally, time-matched control experiments were performed to confirm that the Phe-induced precontractions remained stable throughout the experimental period.

### 2.2. Statistical analyses

Data are presented as mean ± S.E.M. In all experiments, “n” indicates the number of rats studied. Relaxation responses to Ach, SNP, atorvastatin and rosuvastatin are expressed as the percentage of Phe-induced precontraction in that aortic ring. The sensitivities of aortic rings to statins are expressed as the effective concentration required to produce 50% of the maximum response (EC_50_), which was calculated for each concentration-response curve by using probit analysis. Maximum relaxation responses are reported as E_max_ (%), while EC_50_ values are expressed as −log M (pEC_50_). Statistical analyses were performed by using two-way analysis of variance (ANOVA) followed by Bonferonni post-hoc tests. A p-value <0.05 was considered statistically significant. GraphPad Prism software (version 9.4.0, Windows, Boston, Massachusetts, USA) was used for all statistical analyses.

### 2.3. Drugs and chemicals

The drugs used in this research were obtained from Sigma Chemical Co. (USA). Atorvastatin and rosuvastatin were donated by World Medicine (Türkiye). They were dissolved in DMSO, and the final concentration of DMSO in the organ bath was <0.1%. Ach was dissolved in 0.001 N HCl, while all other drugs were dissolved in distilled water and subsequently diluted with fresh Krebs-Ringer solution on the day of each experiment.

## 3. Results

### 3.1. Relaxant responses to Ach and SNP

**Figure 1:**
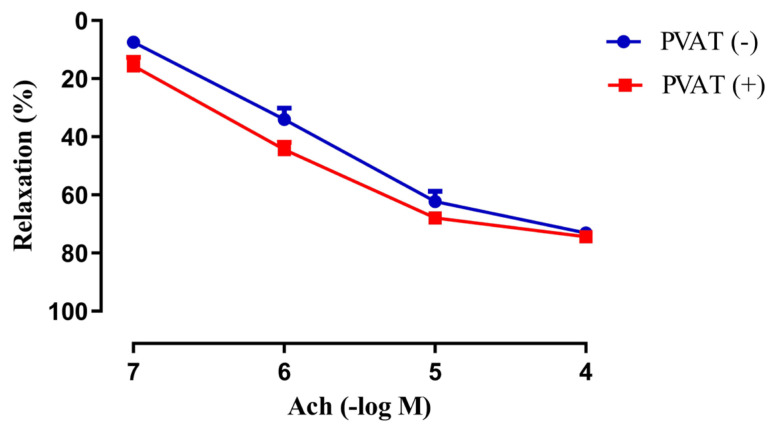
Concentration-dependent relaxant effects of Acetylcholine (Ach, 10^−7^–10^−4^ M) on isolated rat aortic rings **with intact PVAT (+)** and **without PVAT (**−**)** which were precontracted submaximally with Phenylephrine (Phe, 10^−6^–3 × 10^−5^ M) (n = 9–13).

Ach (10^−7^–10^−4^ M) produced concentration-dependent relaxations on isolated rat aortic rings with intact PVAT (+) and without PVAT (−) (Figure 1). The maximal relaxation responses to Ach were determined to be 74.38% ± 1.42% (n = 9) in rings with intact PVAT (+) whereas 73.06% ± 1.86% (n = 13) in rings without PVAT (−). No difference was found in maximum responses or the sensitivity (pEC_50_ values: 5.95 ± 0.15; 5.79 ± 0.11) to Ach between PVAT (+) and PVAT (−) groups, respectively (p > 0.05).

In addition, the directly acting endothelium-independent vasodilator SNP (10^−6^ M) produced similar maximum relaxations in isolated rat aortic rings with intact PVAT (+) (106.25% ± 2.03%, n = 9) and without PVAT (−) (102.45% ± 1.17%, n = 13) (p > 0.05).

### 3.2. Effects of atorvastatin and rosuvastatin on rat aortic rings with PVAT (+) and without PVAT (−)

**Figure 2:**
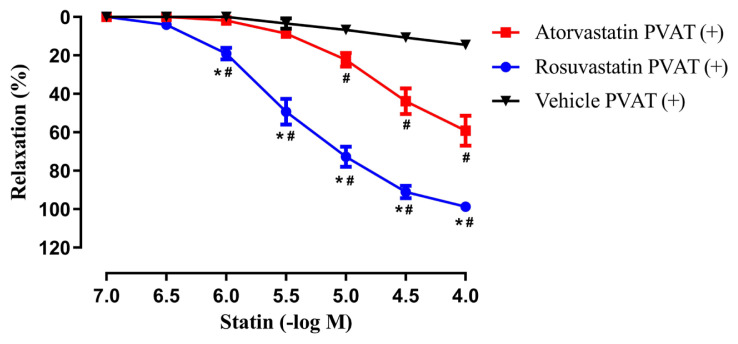
Effects of atorvastatin (10^−7^–10^−4^ M), rosuvastatin (10^−7^–10^−4^ M) and the vehicle (DMSO) on isolated rat aortic rings **with intact PVAT (+)** which were precontracted submaximally with phenylephrine (Phe, 10^−6^– 3 × 10^−5^ M). ^*^p < 0.001, Rosuvastatin PVAT (+) vs. Atorvastatin PVAT (+); **^#^**p < 0.001, Rosuvastatin or Atorvastatin PVAT (+) vs. Vehicle PVAT (+), two-way ANOVA and Bonferroni post-hoc test (n = 6–8).

**Figure 3:**
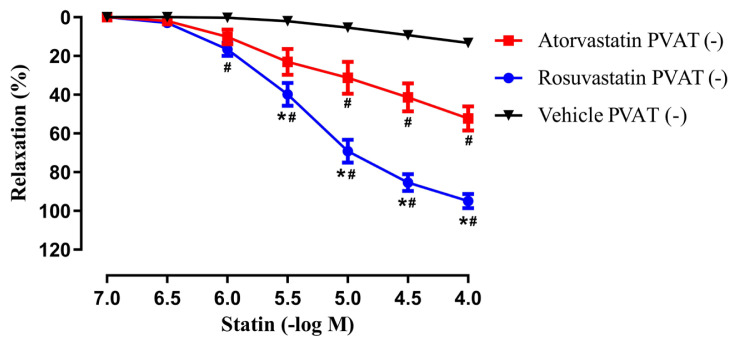
Effects of atorvastatin (10^−7^–10^−4^ M), rosuvastatin (10^−7^–10^−4^ M) and the vehicle (DMSO) on isolated rat aortic rings **without PVAT (**−**)** which were precontracted submaximally with phenylephrine (Phe, 10^−6^ –3 × 10^−5^ M). ^*^p < 0.001, Rosuvastatin PVAT (−) vs. Atorvastatin PVAT (−); **^#^**p < 0.001, Rosuvastatin or Atorvastatin PVAT (−) vs. Vehicle PVAT (−), two-way ANOVA and Bonferroni post-hoc test (n = 7–12).

As shown in Figures 2 and 3, atorvastatin (10^−7^–10^−4^ M) and rosuvastatin (10^−7^–10^−4^ M) produced concentration-dependent relaxations on isolated rat aortic rings with intact PVAT (+) and without PVAT (−) which were precontracted submaximally with Phe (10^−6^–3 × 10^−5^ M).

The maximal relaxation responses to rosuvastatin were determined to be greater than that of atorvastatin in rings with intact PVAT (+) and without PVAT (−). Additionally, a significant difference was found in the sensitivities (pEC_50_) of rosuvastatin compared to atorvastatin in rings with intact PVAT (+) (Table). Moreover, maximum relaxations as well as the pEC_50_ values determined for either atorvastatin or rosuvastatin were found comparable between aortic rings with intact PVAT (+) and without PVAT (−) (p > 0.05) (Table).

On the other hand, the vehicle DMSO, did not produce an important effect on the precontractile tone in rings with intact PVAT (+) and without PVAT (−) (14.45% ± 3.83%; 13.25% ± 3.83%, respectively, n = 7–11) (Figures 2–3).

## 4. Discussion

Atorvastatin and rosuvastatin, which are clinically preferred and potent statins, have been demonstrated to induce relaxation in various vascular preparations [6,10**–**13]. However, the possible role of PVAT in modulating the direct vasorelaxant effects of statins are not investigated so far. The present study originally demonstrated that atorvastatin and rosuvastatin displayed concentration-dependent relaxant effects in rat aortic rings with intact PVAT (+) and without PVAT (−), suggesting that PVAT did not modify the acute vasorelaxant effects of these statins under healthy conditions. Notably, rosuvastatin produced a significantly greater maximum relaxation than atorvastatin in rat aortic rings, regardless of the presence or absence of PVAT.

Several in vitro studies using isolated vessel preparations have demonstrated that statins, including atorvastatin and rosuvastatin, induce acute relaxant effects via endothelium-dependent and -independent mechanisms, beyond their lipid-lowering properties [6–15]. Endothelial vasodilator mediators, including nitric oxide (NO) and/or prostaglandins, which are well known modulators of vascular function, have been shown to mediate the direct vasorelaxant effects of statins [6,10,13,15]. PVAT is also recognized as a key player in the paracrine modulation of vascular function due to its close proximity to the vascular system. By releasing various vasoactive substances, such as adipokines, hydrogen sulfide (H_2_S) and NO, PVAT exerts an anticontractile effect on spasmogen induced contractions and regulates vascular smooth muscle tone [16–19]. Considering the increased evidence regarding the contribution of PVAT in the regulation of vascular homeostasis, herein, we evaluated the possible modulatory role of PVAT in the direct vascular effects of atorvastatin and rosuvastatin.

Previous research focused on the relationship between statins and PVAT function, demonstrating that statin treatment augments the anticontractile effect of PVAT under healthy and disease conditions. In relation, long term (26 weeks) treatment with atorvastatin (50 mg/kg/day) has been shown to restore the anticontractile effect of PVAT in spontaneously hypertensive rats (SHR) [21]. Likewise, a 3-week treatment of Wistar rats with atorvastatin (40 mg/kg/day) was reported to enhance the anticontractile effect of PVAT on Phe induced contractions by stimulating H_2_S production [20]. Interestingly, this modulatory effect was not observed with pravastatin treatment in the latter study. In addition to improving the anticontractile function of PVAT, atorvastatin treatment has been shown to ameliorate the inflammatory profile of PVAT in pathological conditions [23–24]. Although the aforementioned in vivo studies support the interplay between statins and PVAT function, in vitro studies demonstrating the modulatory role of PVAT in the direct vascular effects of statins are lacking.

In the present study, we demonstrated that atorvastatin and rosuvastatin produced concentration-dependent relaxations in Phe-precontracted rat aortic rings with intact PVAT (+) or without PVAT (−). Notably, the maximum relaxation response obtained with either statin was similar between aortic rings with intact PVAT (+) and without PVAT (−). To the best of our knowledge, this is the first study demonstrating that PVAT does not modulate the acute vasorelaxant effects of statins namely, atorvastatin and rosuvastatin, in rat aortic rings. Further research will be intriguing to elucidate the impact of PVAT on statin mediated vascular effects under pathological conditions as well.

Notably, our findings also demonstrated that rosuvastatin produced a greater relaxant effect than atorvastatin in rat aortic rings with intact PVAT (+) and without PVAT (−). Although several clinical studies support the comparative efficacy of atorvastatin and rosuvastatin in the prevention of cardiovascular disease [26–27], some clinical evidence suggests differences in their non-lipid pleiotropic effects, particularly in the vascular system. In this context, rosuvastatin treatment was found to be superior to atorvastatin treatment in improving endothelial function and stabilizing atherosclerotic plaques in patients with coronary artery disease [28–29]. Supporting these clinical observations, our findings demonstrate, for the first time, that rosuvastatin exhibited a greater maximum relaxant effect than atorvastatin in rat aortic rings, emphasizing the difference between atorvastatin and rosuvastatin in terms of in vitro vascular reactivity. In addition, the greater maximum relaxant effect of rosuvastatin compared to atorvastatin does not seem to rely on the differences in endothelium-dependent and -independent relaxant capacities of the aortic preparations, as they were found to be comparable in rings with intact PVAT (+) and without PVAT (−).

Despite a recent in vitro study reporting a similar relaxation response to atorvastatin and rosuvastatin in the rat thoracic aorta [12], experimental studies comparing the direct vasorelaxant effects of atorvastatin and rosuvastatin are lacking. Given the hydrophilic nature of rosuvastatin compared to the lipophilic nature of atorvastatin, the differences in vascular relaxant effects observed in the present study may be attributed to their variations in lipid solubility [30,31]. However, previous in vitro studies evaluating the vasorelaxant effect of various statins have shown either no difference between hydrophilic and lipophilic statins [6,12] or a greater relaxation response to lipophilic statins [9]. Considering these conflicting results, as well as the lack of direct experimental data linking lipid solubility to the observed differences in the vasorelaxant effects of atorvastatin and rosuvastatin, further studies evaluating the impact of other mechanisms affecting vascular function, such as NO release and antioxidant properties would be intriguing.

The present study has some limitations to be considered. We examined the acute effects of statins in the absence and presence of PVAT in an in vitro bioassay system. However, the modulatory role of PVAT in vascular effects of statins may be more evident in an in vivo setting following long-term statin treatment. Furthermore, our findings suggest that PVAT does not contribute to the vascular relaxant effects of atorvastatin and rosuvastatin in young rats under healthy conditions. However, it would be valuable to investigate how this effect may be altered in the context of PVAT dysfunction associated with aging and pathological conditions.

In conclusion, this study demonstrated that both atorvastatin and rosuvastatin induced concentration-dependent relaxation in precontracted isolated rat aorta, regardless of the presence or absence of PVAT. Additionally, rosuvastatin displayed a greater vasorelaxant effect compared to atorvastatin in rat aortic rings with and without PVAT. Whether the difference in the direct vasorelaxant effects of these statins, beyond their potent lipid lowering properties, has clinical significance remains to be evaluated in future studies. This study provides novel evidence that PVAT does not significantly influence statin-mediated vasorelaxation under physiological conditions. However, the impact of PVAT on the vasorelaxant effects of statins may be more critical in aging and pathological conditions, which should be further investigated.

## Figures and Tables

**Table:** The maximal relaxation (E_max_) and pEC_50_ values of atorvastatin (10^−7^–10^−4^ M), and rosuvastatin (10^−7^–10^−4^ M) on isolated rat aortic rings with intact PVAT (+) and without PVAT (−) which were precontracted submaximally with Phe (10^−6^–3 × 10^−5^ M).

	PVAT (+)			PVAT (−)		
	E_max_ (%)	pEC_50_	n	E_max_ (%)	pEC_50_	n
**Rosuvastatin**	98.77 ± 1.51*	5.51 ± 0.13*	7	94.94 ± 3.58#	5.38 ± 0.09	11
**Atorvastatin**	59.21 ± 7.72	4.68 ± 0.13	8	52.25 ± 6.07	5.40 ± 0.90	7

* p < 0.001, Rosuvastatin PVAT (+) vs. Atorvastatin PVAT (+);

# p < 0.001, Rosuvastatin PVAT (−) vs. Atorvastatin PVAT (−), two-way ANOVA and Bonferroni posthoc test.
